# Management of a complicated redo giant dissecting aortic aneurysm

**DOI:** 10.5830/CVJA-2016-087

**Published:** 2017

**Authors:** Ibrahim Kara, Alper Erkin, Halil Ibrahim Erkengel,, Kıyasettin Asil

**Affiliations:** Department of Cardiovascular Surgery, Faculty of Medicine, Sakarya University, Sakarya, Turkey; Department of Cardiovascular Surgery, Faculty of Medicine, Sakarya University, Sakarya, Turkey; Department of Cardiovascular Surgery, Faculty of Medicine, Sakarya University, Sakarya, Turkey; Department of Radiology, Faculty of Medicine, Sakarya University, Sakarya, Turkey

**Keywords:** giant aortic aneurysm, surgical procedures

## Abstract

Giant aortic aneurysm is defined as an aneurysm of the aorta of greater than 10 cm in diameter. This rare condition is associated with a high risk of morbidity and mortality and it may lead to fatal complications such as rupture and/or dissection if not managed with proper surgical planning and expertise. Other than atherosclerosis, the main causes of giant ascending aortic aneurysms include Marfan and Ehlers–Danhlos syndromes. Herein we report on a young male patient who had had an aortic valve replacement five years earlier due to a bicuspid aortic valve leading to aortic failure, accompanied by aortic coarctation. He had an aneurysmal expansion rate of 1.81 cm/year to reach a final aneurysmal diameter of 13.25 cm, which, to our knowledge, represents the largest size ever reported in the literature for such lesions, and in which the redo and aneurysmal wall were adjacent to the sternal margins.

## Introduction

Giant aortic aneurysm is a very rare clinical entity defined as an aneurysm of the aorta that is greater than 10 cm in diameter at its widest point.^1^ The risk of rupture closely parallels the diameter of the aneurysm, with a rupture risk of 31% in lesions in which the diameter of the ascending aorta is greater than 6 cm.^2^ Other main causes of ascending aortic aneurysms include Marfan and Ehlers–Danlos syndromes, in addition to atherosclerosis.^1^ Although the risk of dissection and/or aneurysm is lower in patients with a bicuspid aortic valve (BAV) than in patients with Marfan syndrome, the former condition bears higher clinical significance based on its much higher incidence.^3^

Bicuspid aortic valve is a congenital condition requiring early valvular replacement due to accelerated valvular degeneration. It is a serious disorder of the aorta, frequently co-existing with dilation and rapid expansion of the ascending aorta. Therefore the ascending aorta should be regarded as a component of the pathology, and the type of surgical method to be performed should be carefully assessed in BAV patients requiring surgical intervention.^4^

Here we report on a young male patient who developed a giant ascending aortic aneurysm of 13.25 cm, against a background of the very rare occurrence of a chronic dissection five years after a valvular replacement that had been performed due to BAV failure.

## Case report

**Fig. 1.**
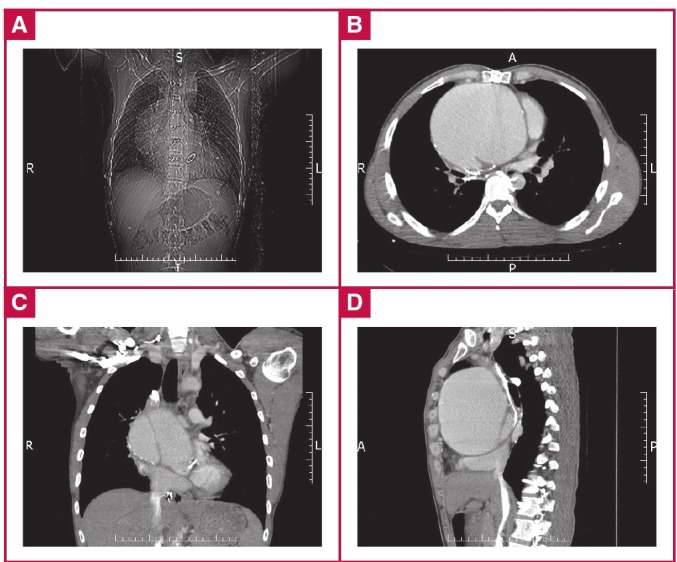
Mediastinal widening seen in pre-operative chest radiography (A). Pre-operative thoracic CT scan: axial (B), coronal (C) and sagittal (D) views showing the giant dissecting aortic aneurysm of 13.25 cm in diameter.

A 33-year-old male patient was admitted to the emergency room with headache, shortness of breath and sleeplessness. A consultation was requested from our unit due to extreme aortic and mediastinal dilatation, seen on chest X-ray (Fig. 1A) as well as a history of open-heart surgery. The patient had had a mechanical aortic valve replacement (23 no St Jude) five years earlier due to BAV failure.

On physical examination, his blood pressure was 132/68 mmHg, heart rate was regular at 82 beats/min and electrocardiography was normal. Chest auscultation was normal but a chest X-ray revealed mediastinal widening and cardiomegaly. His family history was unremarkable.

**Fig. 2.**
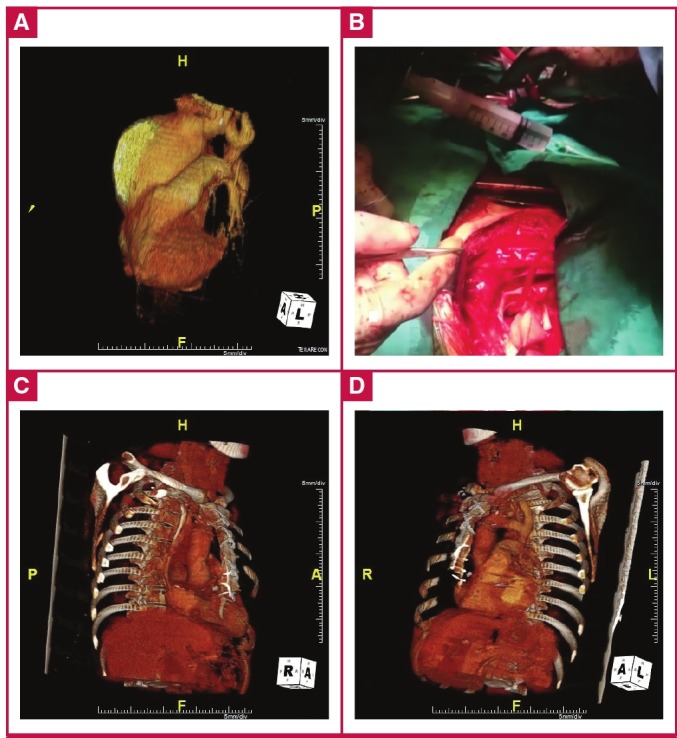
Pre-operative (A) and postoperative (C, D) threedimensional reconstructions from CT scans, and intraoperative (B) view of the aortic aneurysm.

Transthoracic echocardiography (TTE) showed the presence of a mechanical aortic valve prosthesis in its normal anatomical position with normal function, excess dilatation of the proximal ascending aorta (132 mm) and normal left ventricular function. A thoracic contrast-enhanced computed tomography (CT) imaging study revealed a 132.5-mm non-ruptured giant aneurysm and an intra-luminal flap image confined to the ascending aorta (De Bakey type II) that was consistent with dissection (Fig. 1B–D). Surprisingly, despite a normal aortic arch and branches, there was an aortic coarctation just distal to the subclavian artery (Fig. 2A, D).

**Fig. 3.**
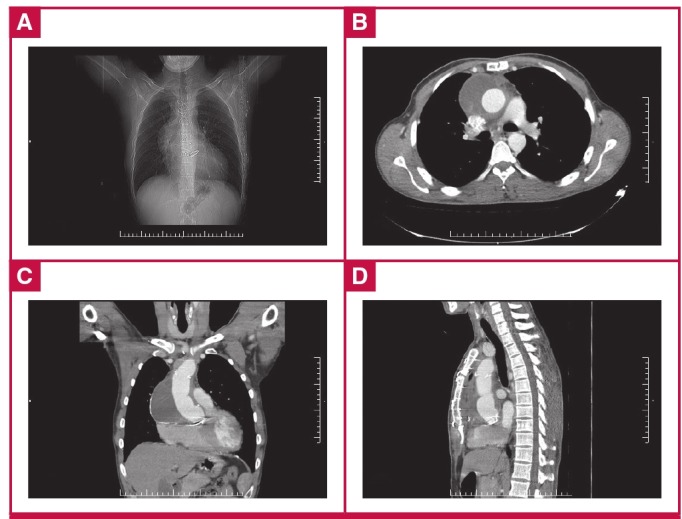
Postoperative radiography (A). Postoperative thoracic CT scan: axial (B), coronal (C) and sagittal reconstructions - mediastinal window view (D).

The patient was haemodynamically and clinically stable and was admitted to our unit for surgery, which consisted of tubular graft interposition in the supracoronary ascending aorta (Fig. 2B–D). He was weaned from mechanical ventilation nine hours postoperatively, and was transferred to the ward on day two. He was discharged uneventfully at day seven. A follow-up thoracic CT at two months showed no problems, and the patient was subsequently followed up in the out-patient unit (Figs 2C, D, 3A–D).

## Surgical technique

As the wall of the ascending aorta was adjacent to the sternum (Fig. 1B, D), arterial cannulation from the right axillary artery was performed, as well as venous cannulation under transoesophageal guidance, from the right femoral vein extending up to the right atrium. Cardiopulmonary bypass (CPB) and cooling was initiated. In such cases, CPB is instituted prior to resternotomy, using deep hypothermia in the event that circulatory arrest is required to gain control of the ascending aorta.

As expected, the aneurysmatic aortic wall adjacent to the sternum was damaged during this procedure. In accordance with the pre-planned surgical strategy, CPB was suspended and deep hypothermia was used for a very short period of time (20°C), during which the aneurysmal sac was rapidly dissected in the distal direction and a clamp was placed at the origin of the aortic arch, which had normal dimensions. CPB was then re-initiated.

Cardiac arrest with hypothermic blood cardioplegia was achieved via hypothermic blood given into the right and left coronary arteries using an osteal cannula, and myocardial protection was provided. Due to the adhesions, cardiac venting was performed through the pulmonary artery. After the body temperature was increased to 27°C, moderate hypothermic circulatory arrest was attained, followed by antegrade selective cerebral perfusion at a flow rate of 10 ml/kg. A selective cannula was then placed into the left main carotid artery to provide bilateral cerebral perfusion. Cranial oxygenation and perfusion were monitored using near-infra-red spectroscopy (NIRS).

After ensuring cerebral protection, a distal aortic anastomosis was performed using no 32 Dacron tubular grafts with the double-Teflon felt technique during moderate hypothermia. A clamp was then placed on the Dacron graft and warming was initiated. Without complete resection of the aneurysmatic aorta from the supra-coronary region, the anastomosis of the proximal aorta was completed with pledgeted 4/0 sutures in the lower half, and the double-Teflon felt technique in the upper half.

After air evacuation and completion of warming, the patient was gradually weaned off CPB. After bleeding was controlled, the patient was de-cannulated and was transferred to the intensive care unit.

## Discussion

This case is of interest from several points of view. First is the asymptomatic clinical course of a giant aneurysm of 13.25 cm with chronic dissection and very high risk of rupture. Second, despite previous reports on several patients with giant aneurysms without rupture, to our knowledge, our patient represents the first asymptomatic redo case of a giant dissecting ascending aortic aneurysm occurring five years after BAV replacement. Third, a surgical treatment of this giant dissecting ascending aortic aneurysm was performed with moderate hypothermic circulatory arrest without any subsequent neurological sequelae in the face of a high risk of aortic injury during sternotomy due to the close proximity to the sternum, caused by the previous aortic valvular surgery.

Giant ascending aortic aneurysms may give rise to very severe clinical complications, among which, dissection and rupture are often fatal.^1^ The risk of rupture is related to the dimensions of the aneurysm and the expansion rate during follow up.^1^ An expansion rate exceeding 1 cm/year or an aneurysmal diameter greater than 6 cm is associated with a dramatic increase in the risk of rupture.^5^,^6^

The risk of dissection in aortic aneurysms is proportional to the increase in diameter, and nearly 25% of patients with chronic aortic dissections may develop aneurysms.^7^ Our patient seemed to have an aortic aneurysm secondary to chronic aortic dissection, based on the fact that the actual underlying pathology was BAV, the aneurysmal expansion rate was high, and the clinical course was of a chronic nature. The expansion rate and risk of rupture in ascending aortic aneurysms in patients with chronic aortic dissection is significantly higher compared to aneurysms on the same site due to other conditions.

The average expansion rate of thoracic aortic aneurysms is 0.1 to 0.2 cm/year.^1^ By contrast, in our patient the ascending aortic diameter was 42 mm at the time of aortic replacement, and this increased to 132.5 mm within a five-year period, which corresponds to a significantly higher rate of expansion than usually reported, namely 18.1 mm/year.^8^ The probable cause of this high rate of expansion was the dissection, which was a complication of a BAV, and the subsequent rapid dilatation of the aneurysm. The lifetime risk of aortic dissection in patients with BAV disease is approximately 6.13%, which is nearly nine times higher than in the normal population.^3^

## Conclusion

Despite the rare occurrence and a very high risk of rupture, giant ascending aortic aneurysms may present with an asymptomatic clinical course, as was the case in our patient, who had a giant aneurysm of 13.25 cm. These challenging aneurysms are adjacent to the sternal wall, require redo operation and are associated with high mortality rates. Therefore adequate surgical planning and expertise are prerequisites for their proper management.
